# Colorectal Cancer Archaeome: A Metagenomic Exploration, Tunisia

**DOI:** 10.3390/cimb45090477

**Published:** 2023-09-19

**Authors:** Nour El Houda Mathlouthi, Hamadou Oumarou Hama, Imen Belguith, Slim Charfi, Tahya Boudawara, Jean-Christophe Lagier, Leila Ammar Keskes, Ghiles Grine, Radhouane Gdoura

**Affiliations:** 1 Laboratoire de Recherche Toxicologie Microbiologie Environnementale et Santé (LR17ES06), Faculté des Sciences de Sfax, Université de Sfax, Sfax 3000, Tunisia; 2 IHU Méditerranée Infection, UMR MEPHI, 19-21, Bd. Jean Moulin, 13005 Marseille, France; 3 Laboratoire de Recherche de Génétique Moléculaire Humaine, Faculté de Médecine de Sfax, Université de Sfax, Avenue Majida BOULILA, Sfax 3029, Tunisia; 4 Department of Pathology, CHU Habib Bourguiba, Sfax 3029, Tunisia; 5 Institut de Recherche pour le Développement (IRD), Aix-Marseille University, Microbes Evolution Phylogeny and Infections (MEPHI), 13005 Marseille, France

**Keywords:** archaeome, metagenomic, gut microbiome, formalin-fixed paraffin embedded tissues, colorectal cancer, *Halobacteria*, *Natrialba magadii*, prognosis

## Abstract

Colorectal cancer (CRC) is a serious public health problem known to have a multifactorial etiology. The association between gut microbiota and CRC has been widely studied; however, the link between archaea and CRC has not been sufficiently studied. To investigate the involvement of archaea in colorectal carcinogenesis, we performed a metagenomic analysis of 68 formalin-embedded paraffin fixed tissues from tumoral (*n* = 33) and healthy mucosa (*n* = 35) collected from 35 CRC Tunisian patients. We used two DNA extraction methods: Generead DNA FFPE kit (Qiagen, Germantown, MD, USA) and Chelex. We then sequenced the samples using Illumina Miseq. Interestingly, DNA extraction exclusively using Chelex generated enough DNA for sequencing of all samples. After data filtering and processing, we reported the presence of archaeal sequences, which represented 0.33% of all the reads generated. In terms of abundance, we highlighted a depletion in methanogens and an enrichment in *Halobacteria* in the tumor tissues, while the correlation analysis revealed a significant association between the *Halobacteria* and the tumor mucosa (*p* < 0.05). We reported a strong correlation between *Natrialba magadii*, *Sulfolobus acidocaldarius*, and tumor tissues, and a weak correlation between *Methanococcus voltae* and healthy adjacent mucosa. Here, we demonstrated the feasibility of archaeome analysis from formol fixed paraffin-embedded (FFPE) tissues using simple protocols ranging from sampling to data analysis, and reported a significant association between *Halobacteria* and tumor tissues in Tunisian patients with CRC. The importance of our study is that it represents the first metagenomic analysis of Tunisian CRC patients’ gut microbiome, which consists of sequencing DNA extracted from paired tumor-adjacent FFPE tissues collected from CRC patients. The detection of archaeal sequences in our samples confirms the feasibility of carrying out an archaeome analysis from FFPE tissues using a simple DNA extraction protocol. Our analysis revealed the enrichment of *Halobacteria*, especially *Natrialba magadii*, in tumor mucosa compared to the normal mucosa in CRC Tunisian patients. Other species were also associated with CRC, including *Sulfolobus acidocaldarius* and *Methanococcus voltae*, which is a methanogenic archaea; both species were found to be correlated with adjacent healthy tissues.

## 1. Introduction

Colorectal cancer (CRC) poses a serious public health problem. Several studies linked the alteration of the intestinal microbiota to colorectal carcinogenesis [1,2,3]. The link between bacteriome and CRC was widely investigated. Bacteria can be pro-carcinogenic directly (via driver bacteria, such as *Fusobacterium nucleatum*) or indirectly (via passenger bacteria, such as *Bacteroides fragilis*) [4]. They may promote intestinal inflammation, leading to a tumor-permissive microenvironment, while inhibiting tumor defense and activating pro-carcinogenic signaling pathways that lead to molecular changes and, therefore, CRC progression via their production of toxins and carcinogenic metabolites [5]. The link between virome and CRC was also investigated. Previous studies highlighted the association between Epstein–Barr virus (EBV), John Cunningham virus (JCV), and human papillomavirus (HPV) and CRC [6,7], and reported their possible involvement in cell cycle disruption and modification of several pathways known to be associated with CRC, such as the Wnt/β-catenin [8]. In addition, increased diversity in the intestinal bacteriophage community in CRC patients was revealed, which can reshape the microbiome, while these viruses can also transit in the epithelial cells of the colon and directly affect tumor growth and its invasiveness [9]. For mycobiome, it was previously found that Basidiomycetes, Ascomycetes, and Malassezia were enriched in the faecal microbiome of CRC patients, whereas Saccharomycetes, such as *Saccharomyces cerevisiae*, were depleted [10]. As for archaea, not many studies were realized. 

Archaea are unicellular extremophile or mesophile micro-organisms [11] that present unique characteristics, such as the lack of peptidoglycan in the cell wall, and a membrane formed by L-glycerol ethers/isoprenoids chains instead of D-glycerol esters/fatty acids [12,13]. They are associated with several pathologies, such as chronic inflammatory bowel diseases, urinary tract infections, and vaginosis [14,15,16]. To investigate its role in CRC, previous metagenomic analyses were performed, which detected archaeal DNA but with a very reduced amount of abundance (0.01 to 17.8% of the total gut microbiome [17]). The composition of gut archaeomes was only revealed in 2020 in a study on the Chinese population using stool from CRC patients [18]. A second paper was published in 2021, presenting the results of the archaeome [19]. 

Other than feces and fresh/frozen tissues, gut microbiome analysis was also performed on formol-fixed paraffin-embedded tissues [20]. This type of specimen is consistently archived and cared for due to its ability to be stored longer at room temperature, which means lower storage costs than frozen tissue [21]. Therefore, they constitute an extensive repository of tissue material usable for long-term clinical diagnostics [22]. However, the use of FFPE tissues had several technical challenges, such as high content of human genomic DNA, low bacterial DNA [22], and sequence artifacts in the DNA extracted [23]. 

Here, we aim to exclusively study the composition of gut archaeomes in Tunisian CRC patients by sequencing and comparing 35 pairs of FFPE tissue samples from tumors and adjacent healthy mucosa to identify archaeal biomarkers of CRC for prognostic interest. 

## 2. Materials and Methods

### 2.1. Human CRC Tissue Specimens

Tissues were obtained from the biobank of the Department of Pathology, Habib Bourguiba Hospital, Sfax, Tunisia, and tissue sampling was approved by the ethics committee of the Ministry of Health in Tunisia. All samples were handled exclusively under strictly anonymized conditions. The study population consisted of 35 cases of colorectal carcinoma retrospectively selected over 3 years from 2020 to 2022. The inclusion criteria for patient selection were as follows: (1) histologically proven adenocarcinoma of CRC, and (2) surgically treated for the first time for CRC. The exclusion criteria were as follows: (1) prior history of other cancers, and (2) prior radiotherapy or chemotherapy treatment. The study design included two samples per patient, consisting of paired tumor tissue (*n* = 35) and normal colorectal mucosa (*n* = 35), which was taken from a distance of at least 10 cm from the tumor, fixed in 10% neutral buffered formalin, and embedded in paraffin. The CRC stages were assigned according to TNM classification (Supplementary Data S1). The localizations of CRC taken for histology fixing are provided in Supplementary Data S1.

A total of 5 serial cuts of 10 microns per sample trimmed of excess paraffin were obtained from paraffin blocks using a microtome, placed in sterile screw-cap microtubes, and stored at room temperature until use.

### 2.2. DNA Extraction and Library Preparation

We realized two protocols for DNA extraction. The first protocol consisted of using a commercial kit: Generead DNA FFPE kit (Qiagen, Germantown, MD, USA). The extraction of DNA from 70 FFPE tissues was performed according to the manufacturer’s instructions. For the second method, two specimens were excluded due to a lack of material (i.e., no more tissues in the blocks). Here, we introduced some modifications to a simple protocol described previously [24]. Briefly, two sections (2 × 10 μm) were stirred and heated at 90 °C for 10 min on a thermocycler with 100 µL of 0.5% Tween-20, before being cooled to 55 °C. Next, we added 2 μL of 10 mg/mL of proteinase K (Sigma, Poole, Dorset, UK) to each tube and set up the thermocycler to 55 °C for 3 h, with gentle shaking every hour. In total, 100 μL of 5% Chelex-100 (Bio-Rad Laboratories, Hercules, CA, USA) in Tris—EDTA was added and heated at 99 °C for 10 min. The tubes were gently shaken and spun at 10,500× *g* while hot for 15 min. To make sure that there were no paraffin residue in the tubes, the samples were spinned at 10,500× *g* with the temperature set up at −8 °C, meaning that any remaining paraffin would be solidified and need to be removed. The samples were heated to 45 °C and 100 µL of chloroform was added. After gentle shaking and additional centrifugation at 10,500× *g* for 15 min, the upper phase (100–150 μL) was retained and stored at −80 °C until use. 

DNA was quantified using a Qubit 3.0 fluorometer (Thermo-Fischer, Waltham, MA, USA). Paired-end libraries were prepared on DNA extracted via the Chelex method using an Illumina Nextera XT library preparation kit (Illumina, San Diego, CA, USA) following the standard protocol.

### 2.3. Sequence Technology and Processing

Sequencing was performed using an Illumina Miseq (Illumina, San Diego, CA) for 2 × 250 paired-end reads. The analysis of reads generated via the sequencer was performed using Galaxy Europe “https://usegalaxy.eu/” (accessed from 4–28 November 2022). The initial quality assessment of the 112,056,964 reads generated was performed using FastQC (Galaxy Version 0.71). Low-quality reads were removed using the Cutadapt tool following the instructions of the tutorial provided by the Galaxy Training Network [25]. Briefly, adapter sequences were trimmed from the 3’ ends of paired-end reads, and reads shorter than 20 bp were discarded. The quality cutoff was set to 20, which allowed for the cutting of low-quality reads from the 5’ and/or 3’ ends of each read before removing the adapter.

### 2.4. Taxonomic and Statistical Analysis

Taxonomic classification of NGS data was performed using Kraken Pipeline, which is highly accurate software designed to assign taxonomic labels to metagenomic DNA sequences using a k-mer based approach to classify sequences, in which short sequences of fixed length (k-mers) are compared to those in the reference database to identify the most likely source organism [26]. Reads were mapped against the partial database “Archaea 2020” using Galaxy Europe “https://usegalaxy.eu/”(accessed from 4–28 November 2022). Before analyzing the output of Kraken, the second step of filtering was performed using Microbiomeanalysist “https://www.microbiomeanalyst.ca/” (accessed from 1–5 December 2022). This method consisted of setting the minimum number of counts at four and the prevalence at 20% of samples to eliminate taxonomic features that were likely caused by sequencing errors or low-level contamination (false positives). The percentage of removed features with low variance was settled at 10% based on the inter-quantile range (IQR) used to remove features that are constant across all conditions of the experiment and are unlikely to be associated with the conditions under study. The filtered data were then normalized via the cumulative sum scaling (CSS) method to correct the differences in sampling depth (library size). A correlation analysis was performed to determine the association between the different archaeal taxa and CRC tissues. A *p*-value of less than 0.05 was considered statistically significant. Statistical analyses were performed using the statistical software package SPSS 20.0. (SPSS 20.0 for Windows; SPSS Inc., Chicago, IL, USA).

## 3. Results

### 3.1. Cohort Description

A total of 68 samples from 35 CRC patients surgically treated between 2020 and 2022 were collected and sequenced. Supplementary Data S1 lists the patients with sample identification, tissue type (tumor or normal mucosa), patient gender and age, tumor location, and TNM classification. Patients in the study included 20 females (57%) and 15 males (43%), with an average age of 63.79 ± 12.7 years, whose ages ranged from 28 to 86 years. The majority of biopsies were taken from the sigmoid colon (*n* = 11 representing 31.4%) and left colon (*n* = 8 representing 22.9%), compared to the remaining locations: caecum (*n* = 6 representing 17%), rectum (*n* = 5 representing 14.3%), colon transverse (*n* = 3 representing 8.6%), and right colon (*n* = 2 representing 5.7%). Histologically, all the samples were described as adenocarcinomas. 

### 3.2. Extraction and Sequencing Outcome

Using the Chelex approach, the mean quantity of DNA extracted from each sample was 19.37 ± 11.4 ng/µL, with a maximum and minimum amount of DNA obtained around 47 ng/µL and 3.14 ng/µL, respectively. The DNA extracted using the Generead kit was much lower than that obtained using the Chelex protocol, with an average of 9.3 ± 11.2 ng/µL; the maximum amount of DNA extracted was 46.2 ng/µL (Supplementary Data S2). However, the DNA extracted from three specimens was undetectable (<0.50 ng/µL), and five other samples showed a DNA quantity lower than 0.2 ng/µL. Due to the insufficient amount of DNA extracted using the Generead kit, we only used DNA obtained with the Chelex method for sequencing. The sequencing of this DNA generated 20.6 Gb of data, with more than 112 million reads and an average of 1,647,896,529 reads per sample. 

After trimming and mapping against a partial archaeal database, 372,385 archaeal reads were found, representing 0.33% of all the reads generated, and then trimmed with an average of 5776.23 archaeal reads per sample. One specimen—Mic115—was lost due to the low number and variability in taxonomic features detected. 

### 3.3. Taxonomic Analysis of Archaeome in CRC Patients

The taxonomic data generated via Kraken (Supplementary Data S3) were normalized and filtered based on a low count and low variance filter (Supplementary Data S4). Our analysis revealed the presence of three main archaeal phyla in adjacent and tumoral tissues: Euryarchaeota, with an abundance percentage of 82.5% in adjacent tissues and 81.02% in tumor tissues; Crenarchaeota, presenting 13.19% of archaeal sequences in adjacent tissues and 14% in tumor tissues; and Thaumarchaeota, showing an abundance rate equal to 4.25% in adjacent tissues and 4.9% in CRC tissues (Supplementary Data S4).

For the archaeal class analysis, three taxonomic features were removed based on prevalence and IQR during the filtering step. The abundance percentage was almost the same in the adjacent tissue and tumor tissues for *Thermococci* (0.78% and 0.71%), *Methanococci* (0.59% and 0.52%), Methanomicrobia (10.86% and 10.91%), *Thermoprotei* (14.74% and 14.60), and *Nitrososphaeria* (4.51% and 4.95%). However, we noticed a decrease in the abundance of *Methanobacteria* in tumor tissues (33.85%) compared to adjacent healthy tissues (37.12%), as well as an increase in the abundance of *Halobacteria* (from 31.37% in adjacent tissues to 34.41% in tumor tissues) (Supplementary Data S4). Spearman’s analysis revealed a significant correlation between *Halobacteria* and tumoral tissues (Table 1). At the order level, we noticed a decrease in the abundance of methanobacteriales from 39.32% in adjacent tissues to 33.33% in tumor tissues. In contrast, natrialbales were more enriched in tumor tissues (33.27% vs. 28.7% in adjacent tissues) (Supplementary Data S4). Correlation analysis revealed a significant association between thermococcales and adjacent healthy tissues (Supplementary Data S5).

We analyzed the archaeal families in the gut microbiome of CRC patients and observed a depletion of *Methanobacteriaceae* with an abundance rate of 35.2% in healthy mucosa vs. 32.5% in CRC tissues, as well as enrichment of *Natrialbaceae* in tumor tissues (30.4% in adjacent tissue vs. 33.6 in CRC tissues) (Supplementary Data S4). Several methanogens and halophile genera were detected in CRC and adjacent tissues. The most abundant was *Methanothermobacter*, which showed a decrease in its abundance rate from 31.12% in adjacent tissues to 28.63% in tumoral tissue, unlike *Halovivax*, which was enriched in CRC tumoral tissues (27.27% in CRC tissues vs. 24.5 in healthy mucosa) (Supplementary Data S4). We reported a significant correlation between *Thermococcus* and *Methanobacterium* and non-tumoral tissues (Supplementary Data S5). The majority of species had similar abundance percentages, except *Methanothermobacter wolfeii*, which was detected in 31.08% of the totality of archaeal reads in adjacent tissues before dropping to 28.29% in the tumor tissues. We also noticed an association between *Methanococcus voltae* and healthy mucosa. For the halophiles, we noticed an increase in the abundance of *Halovivax ruber* from 24.57% in healthy mucosa to 26.9% in CRC tissues (Supplementary Data S4); a significant correlation was highlighted between *Natrialba magadii* and CRC tissues. Moreover, *Sulfolobus acidocaldarius* and *Methanobacterium formicicum* were significantly correlated with tumoral mucosa (*p* < 0.05) (Supplementary Data S5).

## 4. Discussion

In Tunisia, 1657 new cases were registered in 2018, presenting the fourth most common cancer in terms of incidence (Age-standardized incidence rate (ASR) per 100,000 = 11.9) and the third in terms of mortality (ASR per 100,000 = 6.6) [27]. ASR increased significantly from 6.4/100,000 in 1994 to 12.4/100,000 in 2009 [28]. This increase can be explained by the adoption of negative elements of a Westernized lifestyle, such as obesity, physical inactivity, and poor dietary habits [29,30]. Previous studies established a link between this lifestyle and the alteration of the microbiota, thus promoting colorectal carcinogenesis [2,3]. Although metagenomic data on CRC patients’ microbiomes were widely generated and compared to healthy subjects, no metagenomic data were available on gut microbiomes from Tunisian CRC patients. This problem occurs because the new generation sequencing (NGS) techniques are expensive, while the resources available to fund them remain limited in several low- and middle-income countries [31]. The collection of stool samples and fresh tissues may be difficult and expensive; in response, other types of samples were previously proposed for the generation of metagenomic data that do not require a high cost of sampling and storage, such as formalin-fixed paraffin-embedded tissue (FFPE) [20]. In the present study, we propose a simple, inexpensive, and effective methodology to analyze, for the first time, the gut microbiota of Tunisian CRC patients, in particular, the gut archaeome from FFPE tissues, revealing its association with colorectal carcinogenesis. We extracted DNA from collected samples using two protocols: Chelex and a commercial kit (Generead, Qiagen). By comparing the amount of DNA extracted with these two methods, we were able to extract a sufficient amount of DNA to perform Miseq sequencing for all specimens (68 tissues) only when using the Chelex approach, since the extraction using Generead failed to extract 0.2 ng/µL of DNA from eight samples. The use of Chelex for DNA extraction from FFPE tissues was proposed previously and shown to be sufficient to perform PCR [24]. A previous study, using the commercial kit (Generead) for DNA extraction, successfully sequenced all their specimens; however, no amount of extracted DNA was mentioned in this article [16]. Since we followed the kit manufacturer’s instructions for removing excess paraffin from the sections until the final elution step, the kit manual suggests that the presence of little or no DNA in the eluate may be explained by a tissue fixation time that exceeds 20 h. Taking into consideration the reduced amount of DNA extracted, we used Miseq technology for sequencing since it only exaggerates a final DNA concentration equal to 0.2 ng/µL. The generated reads were categorized into high- and low-quality reads; therefore, we performed a “trim and filter” step to improve their qualities. Bioinformatic and statistical analyzes were performed using user-friendly methods, such as Galaxy, Microbiomeanalysist, and SPSS. In addition to being a user-friendly tool, Galaxy allows for the creation and sharing of reproducible analysis workflows; has a diverse set of bioinformatics tools, including those specifically designed for metagenomic analysis; is scalable; and provides built-in support for collaboration. It also allows users to perform complex analysis pipelines without requiring programming or command-line experience [32,33]. As for MicrobiomeAnalyst, it makes it possible to perform complex analyzes without requiring expertise in programming or bioinformatics. It also offers a wide range of analysis pipelines, including taxonomic and functional profiling, differential abundance analysis, network analysis, and machine learning, enabling in-depth analysis of metagenomic data. It provides powerful visualization tools, including heatmaps, bar charts, and network graphs, to explore and interpret data [34,35]. Overall, Galaxy and MicrobiomeAnalyst present a complementary duo for performing an in-depth and comprehensive analysis of metagenomic data and are suitable for both novice and advanced users.

While the link between bacteriome and CRC was first investigated in 2011 [36]; virome [9], mycobiome [10], and archaeome [18] were only studied separately in 2019, 2018, and 2020, respectively. Since 2020, and up to the time of writing this article, only two articles investigated gut archaeomes in CRC patients [18,19]; this problem can be traced back to its difficulties in culture, detection, and identification [37,38]. To overcome these issues, metagenomic analyses were performed to identify the composition of the human gut archaeome. 

Here, our taxonomic analysis revealed the presence of three major archaeal phyla in our specimens: Euryarchaeota, Crenarchaeota, and Thaumarchaeota; these results confirmed the previous findings of a study performed on the stool of CRC and healthy subjects [18]. At the class level, we reported a decrease in methanogens and a significant increase in *Halobacteria* (*p* = 0.049) in tumor tissues. Our results are in agreement with a study carried out on the stool of Chinese CRC patients [18]. While *Halobacteria* are extremely halophilic archaea that can adapt to a wide range of salt concentrations, increased salt intake could explain their detection in the human gut. In Tunisia, a recent study carried out on 194 Tunisian adults showed that their exposure to high doses of salt consumption was equal to 8.1 ± 2.7 g/d [39]; the quantity recommended by the WHO does not exceed 5 g/d [40]. We also highlighted an enrichment of the Thermococcales order, especially the *Thermococcus* genus in healthy mucosa, compared to tumoral tissues, whereas the *Natrialba* genus, which is a haloarchaea, was associated with tumoral tissues. *S. acidocaldarius* and *N. magadii* were correlated with CRC tissues. These associations were not previously reported, unlike the depletion of *M. voltae* in the CRC mucosa, which was highlighted in a previous study [18]. As for *M. formicicum*, its association with gastrointestinal diseases in animals and humans was previously revealed [41,42].

Archaea were not detected in FFPE before, only being found in fresh frozen tissues with an abundance amount equal to 0.2% [16]. The present study encourages analysis of the intestinal archaeome using formalin-fixed paraffin-embedded tissues with a simpler DNA extraction method (using the Chelex method instead of a commercial kit). 

Since we still do not understand the link between the archaeome and colorectal cancer, archaea could affect the rest of the microbiome, contributing indirectly to the initiation of colorectal carcinogenesis. A previous study revealed that archaea enriched in CRC showed both mutualistic and antagonistic interactions with bacteria enriched and depleted in CRC, respectively, suggesting that they may collaborate to promote colon carcinogenesis [18]; therefore, other studies are recommended to understand the interactions between archaea and the totality of the microbiome and its relation to colorectal carcinogenesis. While several previous studies discussed the progress made in creating treatment techniques for CRC, such as the dose personalization of regorafenib and its impact on maximizing patient outcomes [43], the prevention of early death by applying immune checkpoint inhibitors (ICI) in combination with other agents [44], and the combination of cetuximab and chemotherapy and its role in improving overall survival and progression-free survival in patients with metastatic colorectal cancer [45], gut microbiome was recognized as having the potential to impact treatment response in multiple ways: drug metabolism, immunomodulation, inflammation, and tumor microenvironment [46,47,48]. Therefore, the taxonomic analysis of the gut microbiome remains a key factor not only to understand its role in the initiation of colorectal carcinogenesis, but also to provide adequate, personalized, and efficient treatment. The field of archaeome composition in colorectal cancer (CRC) is expected to expand significantly, with an increased focus on research, advances in sequencing technologies, functional characterization of archaea, integration of multi-omics and potential clinical implications, and emerging therapeutic interventions. These developments will deepen our understanding of the role of archaea in CRC and pave the way for personalized diagnostic and therapeutic strategies. 

The main limitations of the study are the small number of samples (*n* = 68) and the absence of comparison between fresh and FFPE tissues. Another limitation is the staining of *Halobacteria* in the tissue sections due to the lack of material. To compensate for this shortage, we worked with FFPE archived and already used in the clinical anatomopathological examination.

## 5. Conclusions

Our study demonstrates that metagenomic analyzes could be performed on FFPE tissues using the Chelex approach for DNA extraction. Our method generated, for the first time, archaeal sequences from FFPE tissues, the bioinformatics analyses performed on which revealed enrichment in *Halobacteria* in tumor tissues compared to healthy mucosa in Tunisian patients with CRC. 

## Figures and Tables

**Table 1:** Correlation analysis of archaeal classes and CRC tissues.

	*p* Value	Spearman Correlation Coefficient *r*
Halobacteria	0.049426	0.24103
Methanococci	0.14671	−0.17923
Nitrososphaeria	0.16139	0.17305
Thermococci	0.48629	−0.086525
Methanobacteria	0.60186	0.064894
Methanomicrobia	0.65482	−0.055623
Thermoprotei	0.84259	−0.024722

## Data Availability

The sequence data were deposited in the NCBI Sequence Read Archive (SRA) under BioProject number of PRJNA905672.

## Supplementary Materials

The following supporting information can be downloaded at: https://www.mdpi.com/article/10.3390/cimb45090477/s1.
